# Efficacy of an Electronic Cognitive Behavioral Therapy Program Delivered via the Online Psychotherapy Tool for Depression and Anxiety Related to the COVID-19 Pandemic: Pre-Post Pilot Study

**DOI:** 10.2196/51102

**Published:** 2023-12-25

**Authors:** Elnaz Moghimi, Callum Stephenson, Anika Agarwal, Niloofar Nikjoo, Niloufar Malakouti, Gina Layzell, Anne O'Riordan, Jasleen Jagayat, Amirhossein Shirazi, Gilmar Gutierrez, Ferwa Khan, Charmy Patel, Megan Yang, Mohsen Omrani, Nazanin Alavi

**Affiliations:** 1 Department of Psychiatry Faculty of Health Sciences Queen's University Kingston, ON Canada; 2 School of Rehabilitation Therapy Queen's University Kingston, ON Canada; 3 Centre for Neuroscience Studies Queen's University Kingston, ON Canada; 4 Patient and Family Centered Care Kingston Health Sciences Centre Kingston, ON Canada; 5 OPTT Inc Toronto, ON Canada

**Keywords:** mental health, depression, anxiety, cognitive behavioral therapy, online, COVID-19, efficacy, electronic cognitive behavioral therapy, online psychotherapy tool, pandemic, evidence-based treatment

## Abstract

**Background:**

Lockdowns and social distancing resulting from the COVID-19 pandemic have worsened the population’s mental health and made it more difficult for individuals to receive care. Electronic cognitive behavioral therapy (e-CBT) is a cost-effective and evidence-based treatment for anxiety and depression and can be accessed remotely.

**Objective:**

The objective of the study was to investigate the efficacy of online psychotherapy tailored to depression and anxiety symptoms during the pandemic.

**Methods:**

The pilot study used a pre-post design to evaluate the efficacy of a 9-week e-CBT program designed for individuals with depression and anxiety affected by the pandemic. Participants were adults (N=59) diagnosed with major depressive disorder and generalized anxiety disorder, whose mental health symptoms initiated or worsened during the COVID-19 pandemic. The online psychotherapy program focused on teaching coping, mindfulness, and problem-solving skills. Symptoms of anxiety and depression, resilience, and quality of life were assessed.

**Results:**

Participants demonstrated significant improvements in symptoms of anxiety (*P*=.02) and depression (*P*=.03) after the intervention. Similar trends were observed in the intention-to-treat analysis. No significant differences were observed in resilience and quality-of-life measures. The sample comprised mostly females, making it challenging to discern the benefits of the intervention in males. Although a pre-post design is less rigorous than a controlled trial, this design was selected to observe changes in scores during a critical period.

**Conclusions:**

e-CBT for COVID-19 is an effective and accessible treatment option. Improvements in clinical symptoms of anxiety and depression can be observed in individuals whose mental health is affected by the COVID-19 pandemic.

**Trial Registration:**

ClinicalTrials.gov NCT04476667; https://clinicaltrials.gov/study/NCT04476667

**International Registered Report Identifier (IRRID):**

RR2-10.2196/24913

## Introduction

The persistence of the novel COVID-19 pandemic significantly deteriorated the mental health of the global population [1]. Although lockdowns and restrictions are critical in reducing viral transmissions, these public health measures generate feelings of uncertainty, fear, isolation, and loneliness in citizens [2,3]. Compared to the pre-pandemic era, an increased prevalence of stress, anxiety, depression, and suicidal ideations has also been observed [4,5]. The psychosocial implications of the COVID-19 pandemic can result in worsened mental health symptoms in vulnerable persons [6]. For example, individuals who experienced mandatory COVID-19 quarantine were 5 times more likely to have suicidal and self-harm ideations than those who did not quarantine [7]. Specific to Canada, suicidal ideation in adults increased from 2.7% to 4.2% during the pandemic [8].

Moreover, 50% of Canadians reported a worsening in their mental health and 41% reported being anxious [9]. Since the onset of the COVID-19 pandemic, the number of Canadians experiencing high to extremely high anxiety quadrupled and those with high self-reported depression more than doubled [10]. Also of concern, study respondents reported a reduction in the quality and quantity of available mental health support systems [10]. Undoubtedly, alternative mental health services are needed to adequately address the adverse consequences of the pandemic, narrow the gap in mental health inequities, and reduce the pressure on an already burdened health care system [11].

Electronically delivered psychological interventions (e-psychotherapy) are easily scalable, cost-effective, and pragmatic methods of delivering necessary mental health care to a wide range of individuals [12]. Using the most evidence-based psychotherapy, electronic cognitive behavioral therapy (e-CBT) offers flexible and accessible mental health care to people affected by lockdowns and social distancing. The goal of cognitive behavioral therapy (CBT) is to enhance the awareness and modification of maladaptive cognitions and behaviors within a short period. Numerous studies have demonstrated the efficacy of e-CBT in a myriad of implementation styles. Self-help, guided, and asynchronous deliveries have all shown effectiveness in symptom management, with results showing comparable improvements in in-person CBT, medications, and control groups [13-17]. e-CBT has also been shown to improve resilience and quality of life, in addition to depressive symptom management [17]. The predesigned therapy content of e-CBT enables clinicians to disseminate core and standardized elements of the therapy at a considerably faster rate [18-22]. This distinguishing feature of e-CBT can contribute to a broader audience reach and shorten wait times [21].

The efficacy of e-psychotherapy has also been demonstrated in populations affected by the COVID-19 pandemic. In a sample of 670 Swedish adults, a 3-week self-guided e-CBT program reduced dysfunctional worry and anxiety related to the pandemic [23]. Additionally, distant-delivered CBT has shown promise in patients with posttraumatic stress disorder (PTSD) during the pandemic [24]. Stress-related insomnia was also reduced in a sample of 194 individuals who completed a 1-week self-guided e-CBT intervention during the pandemic [25]. Further, a largely self-guided e-CBT program consisting of 6 lessons was associated with significant reductions in anxiety and depression symptoms and psychological distress [26]. The same study observed a 504% increase in the number of monthly e-CBT course registrations during the COVID-19 period compared to the year prior [26].

While these studies have highlighted e-CBT’s benefits in treating pandemic-related mental distress, the efficacy of the programs can vary depending on the degree of mental care provider engagement [27]. As therapist engagement positively influences the efficacy of e-CBT programs, a therapist-guided e-CBT program was postulated to render greater benefits than self-guided programs. Therapist-guided e-CBT can improve patient outcomes while simultaneously lowering costs and increasing care capacity [19,22].

The purpose of this study was to develop and administer an e-psychotherapy program for patients with major depressive disorder (MDD) and generalized anxiety disorder (GAD), affected by the COVID-19 pandemic [28]. The program focused on pandemic-related mental health concerns present in people with MDD and GAD. The 9-week therapist-guided e-psychotherapy was available through a secure, confidential, and cloud-based platform. Patient progress was tracked through the platform. The primary objective of the program was to significantly reduce stress and psychological distress in patients, from pre- to postintervention. Additionally, the effects of the program on quality of life and resilience were investigated.

## Methods

### Study Design

This pilot study had a pre-post single-arm design. Since the efficacy of the e-CBT intervention in mitigating GAD and MDD symptoms has already been established in previous trials [18-22], this study focused on the pre-post effects of participating in the e-CBT program during the COVID-19 pandemic. Moreover, since the trial was conducted at the beginning of the pandemic, it was important to observe the pre-post effects in a pilot study and subsequently use that data to inform a randomized controlled trial (RCT). The intervention was a 9-week care provider-guided e-psychotherapy program that addressed mental health problems related to the COVID-19 pandemic.

### Ethical Considerations

All procedures were approved by the Queen’s University Health Sciences and Affiliated Teaching Hospitals Research Ethics Board (6029910). The online psychotherapy tool (OPTT; OPTT Inc), complies with the Health Insurance Portability and Accountability Act, Personal Information Protection and Electronic Documents Act, and Service Organization Control-2. OPTT only collects anonymized metadata and uses encrypted data on its platform.

### Participants

Participants (n=80) were recruited through referrals from Hotel Dieu Hospital and Providence Care Hospital outpatient clinics in Kingston, Ontario, Canada, and self-referrals from social media and web-based communities. Recruitment occurred from June 2020 to June 2021. Participants who provided informed consent were evaluated by a psychiatrist on the research team through a secure video appointment. During the appointments, the Diagnostic and Statistical Manual of Mental Disorders, 5th Edition (DSM-5) criteria were used to determine MDD or GAD diagnoses. To confirm the diagnosis, the Mini-International Neuropsychiatrist Interview (MINI), version 7.0.2 DSM-5, was also administered by a trained research assistant on the team. Of the participants assessed for eligibility, 14 did not meet the inclusion and exclusion criteria and 7 never began the program after enrollment. As a result, 59 participants commenced the study. Demographic information for all participants who began the program can be found in Table 1.

**Table 1:** Demographic information of participants who began the online program.

Participants		Total (n=59)		Completers (n=38)	Dropouts (n=21)
**Age^a^**					
	n	54	35		19
	Mean (SD)	32.26 (12.67)	37.37 (13.53)		35.47 (15.23)
**Gender^b^, n (%)**					
	Woman	41 (69)	30 (79)		11 (52)
	Man	11 (19)	5 (13)		6 (29)
	Other	2 (3)	0 (0)		2 (10)
	Did not indicate	5 (9)	3 (8)		2 (10)

^a^*t*_52_=–0.471; *P*=.64.

^b^χ^2^_3_=6.76; *P*=.08.

Inclusion criteria included adults residing in Ontario, Canada, between the ages of 18 and 65 years; capacity to consent; ability to speak and read English; a primary diagnosis of GAD or MDD, with symptoms that started or worsened during the COVID-19 pandemic; and consistent and reliable access to the internet. Exclusion criteria included active psychosis, acute mania, severe alcohol or substance use disorder, active suicidal or homicidal ideations, and mental health problems that were secondary to a medical condition. To prevent potential confounds, participants were also excluded if they were receiving or had previously received any form of CBT within the past year.

### Intervention

At the start of the program, participants were assigned to a care provider that was supervised by the lead psychiatrist on the team. All the care providers who had previous training in psychotherapy and before the study commencement were trained by a psychiatrist involved in the study. At a specified date during each of the 9 weeks, the care provider assigned a predesigned therapy module to the patient through OPTT, a secure cloud-based platform. The modules were also followed by homework assignments which were due on a specific day of the week. The average completion time of the weekly modules was approximately 40 minutes. The homework was then submitted through OPTT, and appropriate care provider feedback was provided to the patient. To maintain standardized care and efficiency, the care providers used predesigned session-specific feedback templates to respond to each homework submission (Alavi and Omrani [17]). The feedback focused on praising the participant’s time and effort, summarizing material from the previous sessions, and reviewing, discussing, and evaluating the participant’s homework. If participants had additional questions or concerns for their care providers, OPTT’s secure chat option was available. A more comprehensive description of the treatment, care providers, and procedures was previously described in Alavi et al’s [28] protocol.

The care-provider-guided e-psychotherapy program consisted of a combination of CBT, mindfulness therapy [29,30], and problem-solving–based therapy [31,32]. The modules were designed to be accessible on any device (ie, desktops, cellphones, and tablets) and compatible across multiple browsers. The sessions included multiple animations and examples to retain participant interest and engagement. The therapy’s engaging modules were also customized to reflect common challenges faced by individuals with MDD and GAD during the COVID-19 pandemic. These modules were adapted from previous clinical trials, which used a similar approach to treating depression and anxiety [18-22].

The primary intent of the program was to teach individuals how to identify and change maladaptive thought patterns, behaviors, and emotions. The lessons focused on the effects of the pandemic on mood, the basics of CBT, deep breathing techniques, body scan and meditation, the self-care kit, SMART (Specific, Measurable, Achievable, Realistic, and Timely) goals, thinking errors, the 5-part model [33], and thought records. The first 2 sessions addressed symptoms caused by the fear of illness and concerns about personal safety in the context of the pandemic. The remaining 7 sessions focused on building adaptive coping skills to address the uncertainties of the COVID-19 pandemic and related symptoms of depression and anxiety. A combination of CBT techniques, problem-solving techniques, and mindfulness practices was integrated into the program. Textbox 1 provides further detail on the content of each session.

**An overview of the content covered in each weekly session of the online program.**

**(1) Anxiety and depression during the COVID-19 pandemic**

- Introduces psychotherapy structure and cognitive behavioral therapy concepts and discusses common mental health symptoms while setting expectations for the course. Also provides an overview of the COVID-19 pandemic and how it can affect mental health. SMART (Specific, Measurable, Achievable, Realistic, and Timely) goals are provided as an assignment.

**(2) Cognitive behavioral therapy and deep breathing**

- The 5-part model is introduced along with breathing techniques.

**(3) Body scanning and meditation**

- Different techniques for body scanning and meditation are discussed, along with healthy distractions to use as coping mechanisms.

**(4) Self-care kit**

- Techniques to calm the fight or flight response are discussed. Building a self-care kit with calming sensory items are introduced (see, taste, feel, and hear) along with sleep hygiene.

**(5) Thinking errors**

- The idea of thinking errors is introduced with mental filters, jumping to conclusions, overgeneralization, discounting positives, magnification and minimization, emotional reasoning, and more examples discussed. Tools to identify a thinking error and fix it are presented.

**(6) Thought records and automatic thoughts**

- Cognitive reappraisal strategies are discussed initially and the thought record tool to better help understand feelings are taught. Automatic thoughts and how to identify them are included.

**(7) Evidence**

- Ways to compile evidence that a negative automatic thought is unsupported are discussed, along with strategies to better balance thought patterns.

**(8) Alternative and balanced thinking**

- Producing alternative and balanced thoughts are further discussed with reflection and double standard tools being provided.

**(9) Review**

- A general overview of breathing techniques, body scanning, goal setting, self-care kit, testing thoughts, challenging thinking errors, thought records, and double standard techniques are discussed to summarize the content covered in the program.

The care providers in the study were trained research assistants who were under the supervision of the principal investigator, a clinician-scientist with expertise in e-CBT [18-22]. All homework feedback was reviewed by licensed therapists and the principal investigator before submission. The psychotherapy platform enabled care providers to schedule and assign predesigned modules, homework assignments, and symptomatology questionnaires. Feedback, checkups, questions, and concerns were communicated through the platform’s chat feature. The chat feature was a text-based messaging system operating within the OPTT platform. All sessions were followed by homework assignments that were submitted through the platform, reviewed by the care provider, and followed by personalized written feedback within 3 days of submissions. Predesigned and session-specific feedback templates were used for care providers to write their personalized feedback. Feedback for each session was given weekly by the care provider with additional interactions occurring through the chat feature on OPTT; however, often the participant wished to contact them with a question, comment, or concern. The weekly feedback for homework sessions was structured based on predesigned templates that were then customized to fit each participant’s responses, needs, and situation.

### Outcome Measures

Data from all outcomes were collected at baseline, week 4, and after the last session at week 9. The severity of anxiety and depression symptoms were assessed using the 7-item Generalized Anxiety Disorder Questionnaire (GAD-7) [34] and 9-item Patient Health Questionnaire (PHQ-9) [35], respectively. The 14-item resilience scale (RS-14) [36] was used to measure resilience levels. Scores range from 14 to 98 with scores less than 65 indicating low resilience. Finally, scores from the Quality of Life Enjoyment and Satisfaction Questionnaire (Q-LES-Q) [37] were used to measure the quality of life. Q-LES-Q scores range from 14 to 70. All the questionnaires were collected directly through OPTT. The GAD-7 assesses the severity of symptoms related to anxiety with each response ranging from 0 (not at all) to 3 (nearly every day) with scores ranging from 0 to 21 with a higher score representing more severe symptoms. The PHQ-9 assesses a client’s depressive symptom severity as well as functional health. Each question was scored 0 (not at all) to 3 (nearly every day) with a total score of 0 to 27 with 27 being the most severe. The RS-14 evaluates emotional resilience with each question ranging from 1 (strongly disagree) to 7 (strongly agree). Scores range from 14 to 98 with a higher score equating to increased resilience. The Q-LES-Q assesses levels of enjoyment and satisfaction in daily functioning and life. Each question was rated from 1 (very poor) to 5 (very good) with scores ranging from 14 to 70, with a higher score indicating a better quality of life.

### Statistical Analysis

A 1-way repeated measure ANOVA was conducted to evaluate the differences in clinical scores of study completers at baseline, midtreatment, and at the end of treatment. Independent samples *t* tests were used to compare pre- and posttreatment scores for all clinical outcomes. For this analysis, intention-to-treat analysis was used to include data from participants who did not complete the study in its entirety. Missing data were not imputed and were analyzed on a per-protocol basis. The significance level for all tests was set to α=.05. All statistical analyses were conducted using IBM SPSS Statistics for Mac (version 24; IBM Corp).

## Results

**Figure 1:**
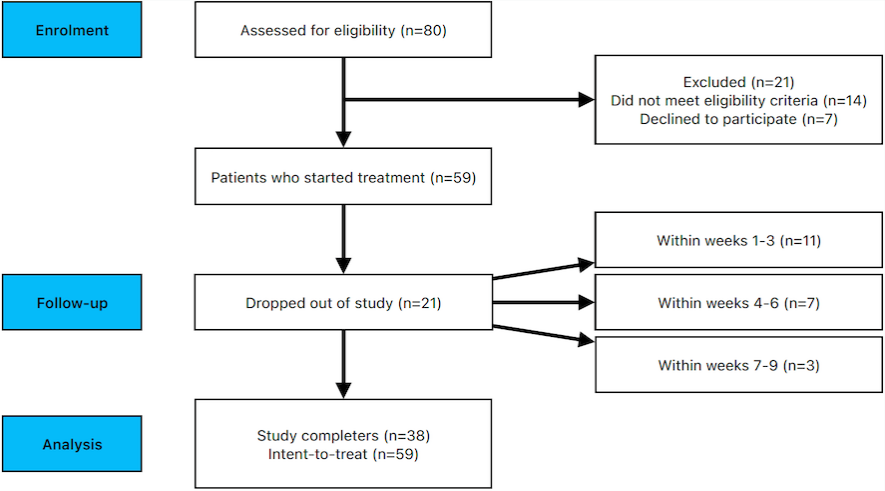
Flow diagram of study recruitment.

Out of the eligible participants who commenced the study (N=59; Figure 1), 21 participants dropped out of the study (n=11 from weeks 1-3, n=7 from weeks 4-6, and n=3 at week 7), and 38 participants completed the study. Reasons for dropout were not disclosed. Most of the total sample identified as women (n=41, 69.49%). A total of 2 participants identified as other and both dropped out of the treatment at weeks 4 and 6, respectively. The average age of the sample was 32.26 (SD 12.67). No significant differences were observed at baseline for any demographic variables (Table 1) or scores of treatment completers and dropouts (Table 2) for participants who began the program. A significant difference was observed between the number of sessions completed by those who dropped out and those who finished the program (*P*<.001). On average, treatment dropouts completed 40.77% of the treatment before dropping out.

GAD-7 scores were reduced from 11.57 to 9.86 to 9.43 (start, mid, and end, respectively). A 1-way repeated measures ANOVA demonstrated improvements in GAD-7 scores (*P*=.02; Table 3). Bonferroni post hoc analysis demonstrated a significant reduction in GAD-7 scores at 0 weeks and 9 weeks (*P*=.02) but not at 0 and 4 weeks (*P*=.22) and 4 and 9 weeks (*P*=.99). Intention-to-treat analysis using unpaired sample *t* tests also demonstrated a significant lowing of scores from pre- to posttreatment (*P*=.02; Table 4).

**Figure 2:**
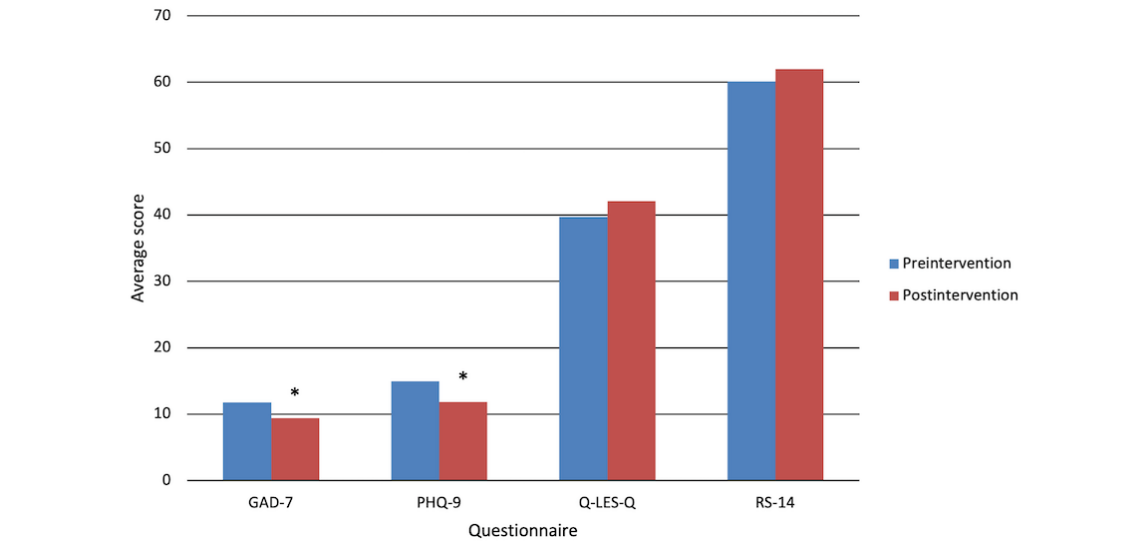
Average intention-to-treat scores (n=38) for each questionnaire, pre- and postintervention. * denotes a significant difference at *P*<.05. GAD-7: Generalized Anxiety Disorder 7-Item Questionnaire; PHQ-9: Patient Health Questionnaire 9-Item; Q-LES-Q: Quality of Life Enjoyment and Satisfaction Questionnaire; RS-14: 14-item resilience scale.

PHQ-9 scores were reduced from 14.65 to 12.43 to 11.84. One-way repeated measures ANOVA demonstrated significant improvements in PHQ-9 scores (*P*=.03; Table 3). Bonferroni’s post hoc analysis demonstrated a significant reduction in GAD-7 scores at 0 weeks and 9 weeks (*P*=.01) but not at 0 weeks and 4 weeks (*P*=.18) and 4 and 9 weeks (*P*=.99). Intention-to-treat analysis using unpaired sample *t* tests also demonstrated a significant reduction in scores from pre- to posttreatment (*P*=.02; Table 4). ANOVA and intention-to-treat analyses indicated no significant differences in Q-LES-Q or RS-14 scores over the 9 weeks (Figure 2).

Regarding noncompleters, 6 participants completed session 1, three completed session 2, two completed session 3, one completed session 4, two completed session 5, four completed session 6, and three completed session 7.

**Table 2:** Demographics and characteristics of the sample.

Characteristics		Total (n=59)	Completers (n=38)	Dropouts (n=21)	Analysis (completers vs dropouts)	
					*t* test (*df*)	*P* value
Sessions completed, mean (SD)		7.10 (2.92)	9 (0)	3.67 (2.35)	–10.39 (20)	≤.001
**Baseline scores, mean (SD)**						
	GAD-7^a^	11.74 (5.15)	11.58 (5.07)	12.05 (5.40)	0.33 (57)	.74
	PHQ-9^b^	14.95 (6.46)	14.53 (6.26)	15.71 (6.90)	0.50 (57)	.67
	Q-LES-Q^c^	39.71 (8.89)	40.05 (8.42)	39.10 (9.86)	–0.39 (57)	.70
	RS-14^d^	60.12 (15.70)	59.45 (15.11)	61.33 (17.03)	0.44 (57)	.66

^a^GAD-7: 7-item Generalized Anxiety Disorder Questionnaire.

^b^PHQ-9: 9-item Patient Health Questionnaire.

^c^Q-LES-Q: Quality of Life Enjoyment and Satisfaction Questionnaire.

^d^RS-14: 14-item resilience scale.

**Table 3:** Means, SDs, and ANOVA of primary outcomes at 3 time points within the 9-week trial.

Questionnaire	Baseline (0 weeks), mean (SD)	Midtreatment (4 weeks), mean (SD)	Posttreatment (9 weeks), mean (SD)	Repeated measures ANOVA (Time)		
				*F* test (*df*)	*P* value	ηp^2^
GAD-7^a^ (n=37)	11.57 (5.14)	9.86 (4.71)	9.43 (5.42)	4.00 (2, 36)	.02	0.10
PHQ-9^b^ (n=37)	14.65 (6.30)	12.43 (7.74)	11.84 (7.41)	3.72 (2, 36)	.03	0.094
Q-LES-Q^c^ (n=36)	40.17 (8.53)	41.64 (9.63)	42.11 (11.79)	0.670 (2, 35)	.51	0.242
RS-14^d^ (n=37)	59.19 (15.23)	62.00 (17.29)	42.83 (19.06)	1.96 (2, 46)	.15	0.052

^a^GAD-7: 7-item Generalized Anxiety Disorder Questionnaire.

^b^PHQ-9: 9-item Patient Health Questionnaire.

^c^Q-LES-Q: Quality of Life Enjoyment and Satisfaction Questionnaire.

^d^RS-14: 14-item resilience scale.

**Table 4:** Means, SDs, and pre-post independent samples *t* test outcomes^a^.

Questionnaire	Baseline (0 weeks)		Posttreatment (9 weeks)			Independent *t* test	
	n	Mean (SD)	n	Mean (SD)	*t* test (*df*)		*P* value (1-sided)
GAD-7^b^	59	11.75 (5.15)	37	9.43 (5.42)	2.100 (94)		.02
PHQ-9^c^	59	14.95 (6.46)	37	11.84 (7.41)	2.170 (94)		.02
Q-LES-Q^d^	59	39.71 (8.89)	36	42.11 (11.79)	–1.052 (59.18)		.15
RS-14^e^	59	60.12 (15.70)	37	62.00 (19.06)	–0.526 (94)		.30

^a^Equal variances are assumed, and all significance is listed as 1-tailed.

^b^GAD-7: 7-item Generalized Anxiety Disorder Questionnaire.

^c^PHQ-9: 9-item Patient Health Questionnaire.

^d^Q-LES-Q: Quality of Life Enjoyment and Satisfaction Questionnaire.

^e^RS-14: 14-item resilience scale.

## Discussion

### Principal Findings

This study aimed to investigate the efficacy of an e-CBT program focused on mental health challenges that were initiated or worsened during the COVID-19 pandemic. A single-arm pre-post study design was used to observe changes in clinical symptoms associated with an e-CBT program disseminated via OPTT at the beginning of the pandemic. The program focused specifically on managing anxiety and depression-related symptoms and navigating through challenges during this time. The findings indicated significant improvements in clinical symptoms after the e-CBT intervention.

Within program completers, anxiety symptoms were reduced by 18.50% and depression symptoms by 19.19%. Average baseline levels of GAD-7 scores indicated moderate anxiety, which was reduced to mild level posttreatment. While average PHQ-9 scores indicated moderate to severe levels of depression, the score moved from the higher end to the lower end of the range posttreatment. Symptoms and disorders related to anxiety and depression are 2 of the most common mental health concerns during the COVID-19 pandemic [5]. Prevalence rates during this period have been reported to be 31.90% for anxiety and 33.70% for depression [5]. Slightly greater reductions of 19.74% in anxiety symptoms and 20.80% in depression symptoms were observed in the intention-to-treat analysis. These findings suggest that treatment dropouts may have also demonstrated some degree of improvement in their clinical symptoms.

Dropout rates for this study were 35.59%, which is on par with rates found in other therapist-guided online interventions and lower than self-guided online interventions [38]. Therefore, the addition of therapists is suggested to reduce attrition rates of online interventions. Although a previous 12-week mobile app effectively improved mental health during the pandemic [39], this study supports the sufficiency of a 9-week intervention in mitigating clinical symptoms. The addition of a care provider to this online program may have improved adherence and sped the trajectory of clinical improvement. Indeed, guided interventions not only demonstrate higher efficacy than their unguided counterparts but can significantly increase completion rates of online interventions targeting depression and anxiety disorders [40]. This study contributes to the body of evidence by suggesting that the time needed for observable clinical improvements may lie between 4 and 9 weeks.

Although improvements in clinical symptoms were observed, there were no significant differences in quality of life and resilience scores before and after the intervention. It is noted that resilience scores can be a screening tool for quality of life, which may partly explain why both scores did not significantly differ pre- and postintervention [36]. Although it requires further investigation, it is postulated that environmental changes may have contributed to a stagnation of the scores [41]. The study was conducted toward the beginning of the pandemic when individuals were just beginning to become acclimated to lockdowns, quarantines, and social distancing laws. There is mounting data that highlight declining mental health as the pandemic persisted [42-44]. At the same time, social isolation, loneliness, financial anxiety, the unknown nature of the virus, collapsed health care systems, high death rates, and other factors inevitably contributed to a worsening of quality of life [45-47]. It is noted that the nonsignificant improvements in quality of life and resilience scores postintervention may have been due to a relatively small sample size. However, program efficacy may be enhanced with the addition of more unique strategies and skills that specifically target these factors during the pandemic.

Additionally, it is possible that changes in quality of life could occur later after program completion, as they may take time to manifest. Future work should incorporate a long-term follow-up to investigate these possible changes. The efficacy of other situation-specific digital psychotherapeutics related to population-wide crises (ie, pandemics and natural disasters) should continue to be developed and further investigated.

### Limitations

Although the study possessed many strengths, it was not without limitations. Instead of an RCT, a pre-post study design was used for several reasons. First, the efficacy of online psychotherapy programs has already been demonstrated in previous trials [18-22]. Second, the study was implemented as a pilot that aimed to explore the efficacy of the e-CBT program over a critical period. This project was used as a pilot and proof of concept to inform a future and larger-scale RCT. It was necessary to implement a design that factored in preintervention scores that coincided with the beginning stages of the pandemic.

Nevertheless, previous data support attributing the improvement symptoms to the online psychotherapy intervention. The second limitation pertained to the gender dispersion within the sample. The sample was relatively homogenous, with 69.49% of participants identifying as women. Differences in clinical outcomes vary due to sex-based differences in immunological response and gender-based differences in behavior and comorbidities [48]. As a result, males tend to experience greater severity and fatality for COVID-19 infections than females [48]. Since the intervention was designed for individuals with mild to moderate clinical symptoms, fewer men may have met the inclusion criteria, thereby resulting in a gender-imbalanced sample. However, it is possible that the program was not as accessible to men, and a qualitative investigation into this should be implemented in the future. Additionally, there is a possible confounder of geographic location affecting symptom severity. As government lockdown restrictions were not always universal across the province, living in a different area of Ontario could have resulted in heightened or lessened restrictions, altering the stressors in that participant’s life.

### Conclusions

In conclusion, the study demonstrated significant improvements in clinical symptoms of anxiety and depression after the use of a 9-week COVID-19–specific e-CBT program. Taken together, e-CBT provides individuals limited by location, time, and cost access to evidence-based and effective therapies. The evidence strongly suggests that online psychotherapy can supplement this care model. Although no changes in quality of life or resilience were reported, these findings may be due to the persistent environmental challenges outside the normative levels observed prepandemic. While the efficacy of e-CBT has been observed across various populations, it is warranted for future studies to investigate the role of gender in treatment availability and help-seeking.

## Abbreviations

CBT: cognitive behavioral therapy
DSM-5: Diagnostic and Statistical Manual of Mental Disorders, 5th Edition
e-CBT: Electronic cognitive behavioral therapy
e-psychotherapy: Electronically delivered psychological interventions
GAD: generalized anxiety disorder
GAD-7: 7-item Generalized Anxiety Disorder Questionnaire
MDD: major depressive disorder
MINI: Mini-International Neuropsychiatrist Interview
OPTT: online psychotherapy tool
PHQ-9: 9-item Patient Health Questionnaire
PTSD: posttraumatic stress disorder
Q-LES-Q: Quality of Life Enjoyment and Satisfaction Questionnaire
RCT: randomized controlled trial
RS-14: 14-item resilience scale
SMART: Specific, Measurable, Achievable, Realistic, and Timely
